# Progress and Challenges in Precise Treatment of Tumors With PD-1/PD-L1 Blockade

**DOI:** 10.3389/fimmu.2020.00339

**Published:** 2020-03-12

**Authors:** Youhai Jiang, Xiaofang Zhao, Jing Fu, Hongyang Wang

**Affiliations:** ^1^Division of Life Sciences and Medicine, Cancer Research Center, The First Affiliated Hospital of USTC, University of Science and Technology of China, Hefei, China; ^2^International Cooperation Laboratory on Signal Transduction, Ministry of Education Key Laboratory on Signaling Regulation and Targeting Therapy of Liver Cancer, Shanghai Key Laboratory of Hepato-biliary Tumor Biology, Eastern Hepatobiliary Surgery Hospital, Second Military Medical University, Shanghai, China; ^3^Cancer Institute, Fudan University Shanghai Cancer Center, Shanghai, China; ^4^National Center for Liver Cancer, Shanghai, China

**Keywords:** PD-1, PD-L1, immunotherapy, patient response, precise treatment

## Abstract

Immune checkpoint inhibitors target the inhibitory receptors on T cells to reinstate their antitumor ability and have shown significant efficacy in treating various cancers. However, because of tumor heterogeneity and many other uncover reasons, the objective response rate for programmed death 1 and programmed death-ligand 1 (PD-1/PD-L1) blockade is only 20 to 30%; its response rate in solid tumors is relatively low, and different degrees of side effects have occurred. There are still many unknown factors affecting the therapeutic effectiveness of PD-1/PD-L1 blockade. Additionally, screening the responding tumor patients accurately and improving the response rate and efficacy are huge challenges for tumor precise treatment. Here, we attempt to summarize the recent progress in response prediction and combined application of PD-1/PD-L1 blockade and briefly discuss the methods and evaluations combined with PD-1/PD-L1 blockade to improve the implementation of precision immunotherapy.

## Introduction

Immune checkpoint inhibitor (ICI) enhances effector T-cell function and has elicited long-term remission in patients with a broad spectrum of tumors. Over the past decade, ICIs have revolutionized the clinical management of advanced malignant tumors and dramatically changed the landscape of cancer treatments that rely heavily on radiotherapy, chemotherapy, and surgical resection. Programmed death 1 (PD-1) is expressed on activated T cells and negatively regulate T-cell responses, acting as a key checkpoint molecule in tumor-induced immune suppression; blockade of the interaction of PD-1 with its ligand, PD-L1 or PD-L2, has been shown to enhance the antitumor activity of T cells (1–4). Antibodies targeting PD-1/PD-L1 checkpoint stimulate the immune system to keep the tumor in check by releasing the immunosuppression. This strategy has emerged as a novel cancer therapy mechanism and plays an increasingly important role in the treatment of serious tumor types.

Although many patients with malignant tumors benefit from immunotherapy using PD-1/PD-L1 inhibitors, there are still no effective predictive biomarkers to guide the clinical precision medicine approach and clinical trial design at present. The research and identification of immunotherapy-related predictive indicators can promote tumor precise treatment and overcome drug resistance or adverse reactions. This review focuses on the recent progress in the efficacy improvement of PD-1/PD-L1 inhibitors and the effective screening of patients to minimize the side effects. Herein, we summarized the biomarkers for predicting efficacy, common side effects and causes, and combination therapy strategies associated with PD-1/PD-L1 therapy.

## Response Prediction and Influencing Factors of PD-1/PD-L1 Blockade Treatment

### Gene Mutations

Genomic alterations in tumor cells correlate with tumorigenesis and response to anti–PD-1 therapy (5, 6). Tumor mutation burden (TMB) is an important indicator for evaluating the immunogenicity of tumors and an emerging biomarker for predicting the response to immunotherapy using cytotoxic T-lymphocyte–associated protein 4 (CTLA-4) and PD-1 checkpoint inhibitors (7–11). A correlation coefficient of 0.74 between TMB and anti–PD-1/PD-L1 immunotherapy response suggests that 55% of the differences in objective response rate (ORR) across cancer types may be explained by TMB (12). Although there may not be one universal definition of a high TMB, tumors with mismatch repair deficiency (MMR-d) and microsatellite instability (MSI) showed remarkable response to ICI therapy (6, 7, 13). Tumors with MMR-d can accumulate thousands of mutations by sequence alterations in the microsatellites, which render the tumors immunogenic and sensitive to ICI therapy (8). The US Food and Drug Administration (FDA) approved the use of pembrolizumab and nivolumab for the treatment of patients with advanced or metastatic solid tumors with MSI-high or MMR-d in 2017.

### PD-1 Ligand Expression

Immune checkpoint inhibitors targeting the PD-1 axis include antibodies directed at PD-1, blocking receptor interaction with its ligands, PD-L1 and PD-L2 (1). Programmed death 1 ligand expression in solid tumors has been reported as a predictive biomarker of benefit from PD-1/PD-L1 axis inhibitors. Programmed death 1 ligand is highly expressed in various tumor cells and myeloid cells (14, 15), and multiple studies on a variety of tumor types have found the PD-L1 overexpression to be associated with ICI response (16–21). Conversely, other studies proved no such association (22, 23). The FDA has approved PD-L1 immunohistochemistry as a companion diagnostic indicator for anti–PD-1 therapy in patients with non–small cell lung cancer (NSCLC) (24–26). Programmed death 1 ligand on the tumor-derived exosomes released from some types of tumor is involved in the immune evasion of tumor cells (27–29). In the preclinical model of colorectal cancer, administration of PD-L1 antibody in combination with the inhibition of exosomal PD-L1 secretion could achieve tumor-suppressive effects (28). Exosomal PD-L1 is also a potential immunotherapeutic target that can address the current resistance to PD-L1 antibody therapy. The level of exosomal PD-L1 can indicate the level of T-cell activation by immunological checkpoint inhibitors and could be used as a prognostic marker (27). PD-L2, the other known ligand of PD-1, positivity significantly predicted clinical response to pembrolizumab (30), while more studies are needed to determine the role of PD-L2 in PD-1 blockade treatment.

### Heterogeneity of the Tumor Immune Microenvironment

The tumor microenvironment is a heterogeneous (31) and immunosuppressive environment composed of different cells and molecules (32, 33). The density, phenotype, and diversity of tumor-infiltrating lymphocytes (TILs) are crucial for ICI response (25). For some types of tumors, the level of TILs is a potential biomarker for ICI response (34). Many other immune cells may also affect the efficacy of anti–PD-1/PD-L1 therapy. The number of stimulatory dendritic cells (SDCs) in human melanoma can predict the patient's immune response and overall survival (OS), and SDC abundance is associated with the cytokines produced by lymphocytes, notably natural killer (NK) cells in human tumors (35). Therefore, the presence of NK cells in tumors is associated with an increase in the number of SDCs and the patient's long-term response to PD-1 antibody. Tumor-associated macrophages (TAMs) are associated with poor anti–PD-1 response in patients with melanoma (36). Although anti–PD-1 antibodies could initially bind to T cells as intended, the TAMs quickly removed these antibodies from T cells, thus inactivating them (37). In summary, a deeper understanding of the heterogeneity of the tumor immune microenvironment is essential to develop sensitive methods or potential predictive biomarkers for providing all the fingerprints of tumor microenvironment and then improve the efficacy of tumor immunotherapy.

### Systemic Effects

#### Obesity

Obesity promotes tumorigenesis and progression associated with increased immunosuppression and potential side effects (38–42). Obesity was also shown to be associated with an increased efficacy of PD-1/PD-L1 blockade in both preclinical and clinical studies involving cancer patients (43). A retrospective study found the correlation between obesity and the efficacy of ICIs in cancer patients; ORR was significantly higher in the overweight/obese patients than in the non-overweight patients (44). Another study demonstrated that obesity is associated with increased efficacy of PD-L1 blockade in both tumor-bearing mice and clinical cancer patients (40). The preclinical trial on breast cancer found that fat cells overexpress PD-L1, affecting the therapeutic effectiveness and outcomes in breast cancer patients, and after inhibiting lipogenesis can selectively reduce the expression of PD-L1 in mouse adipose tissue and enhance the antitumor effect of PD-L1 or PD-1 antibody in a breast cancer model (40). Clinical studies have found that NSCLC patients with body mass index (BMI) ≥25 kg/m^2^ have significantly reduced mortality after receiving the PD-L1 antibody treatment (especially in the case of high PD-L1 expression). However, analysis showed that treatment-related adverse events were not related to BMI (45). There may be other unknown factors affecting the response of obese patients to ICI treatment, such as the function and metabolic status of immune cells, which need to be studied further.

#### Gut Microbiome

Preclinical trials on murine models showed that the microbiome may influence the efficacy of some types of cancer treatment, particularly immunotherapy (46, 47). Researchers analyzing the fecal microbes in responding and non-responding melanoma patients undergoing anti–PD-1 immunotherapy found significant differences in the diversity and abundance of the gut microbiome in the two groups. Further analysis found that the intestinal bacteria in the responding patients' group enhanced systemic and antitumor immunity; meanwhile, germ-free mice receiving fecal transplants from responding patients achieved good tumor regression progress (48). These findings suggest that fecal transplantation may improve the efficacy of PD-1 and drug resistance–related issues in the future.

#### Neutrophil/Lymphocyte Ratio

Neutrophil-lymphocyte ratio (NLR) is a significant prognostic marker in many tumor therapies (49–53); high NLR indicates host inflammation and is associated with shorter OS in several tumor types (49). Comparisons of NLR before and after treatment with PD-1/PD-L1 inhibitors showed that patients with NLR ≥4 were found to have shorter OS and disease progression-free time before treatment (54). The NLR at 6 weeks after treatment initiation was a prognostic marker in patients with advanced NSCLC treated with anti–PD-1 antibody (55). These findings suggest that a high posttreatment NLR alone or its combination with other prognostic biomarkers can be useful indicators of OS and progression-free survival (PFS) in ICI treatment.

## Combination Therapy Strategy

The three most common combination therapy strategies are PD-1/PD-L1 inhibitors in combination with CTLA-4 monoclonal antibodies (mAbs), chemotherapy, and targeted therapies (Table 1). There are four combination strategies approved by the FDA: two for kidney cancer [pembrolizumab (PD-1 antibody) plus axitinib (VEGFR/PDGFR inhibitor), and avelumab (PD-L1 antibody) plus axitinib], one for endometrial carcinoma [pembrolizumab plus lenvatinib (VEGFR/FGFR inhibitor)] and one for NSCLC (atezolizumab, bevacizumab, and chemotherapy) (57).

**Table 1:** Examples of clinical trials for combination therapy strategy.

**Trial, phase (patient no.)**	**Condition or disease**	**Drug**	**Combination**	**Efficacy**	**Highest grade toxicities (%)**	**NCT (reference)**
CheckMate-012 (phase I, *n* = 472)	Advanced NSCLC	Nivolumab	Ipilimumab, gemcitabine, cisplatin, pemetrexed, paclitaxel, carboplatin, bevacizumab, erlotinib	MPFS 12.7 months ORR 47 and 39%	Grade 3–4 adverse events were 42 and 31%	NCT01454102
CheckMate-067 (phase III, *n* = 1,296)	Unresectable or metastatic melanoma	Nivolumab	Ipilimumab	MPFS 11.5 months	Grade 3–4 adverse events 55% four treatment-related deaths: two in the combined group	NCT01844505
CheckMate-227 (Phase III, *n* = 2,220)	NSCLC	Nivolumab	Ipilimumab or nivolumab plus platinum doublet chemotherapy vs. platinum doublet chemotherapy	TBD	TBD	NCT02477826
Exploratory clinical study of apatinib and SHR-1210 in treating advanced hepatocellular carcinoma or gastric cancer (56) (phase I, *n* = 43)	Advanced HCC, GC/EGJC	SHR-1210 (anti–PD-1 antibody)	Apatinib (VEGFR2 inhibitor)	ORR 30.8%;	Dose-limiting toxicity events (26.7%); grade 3 lipase elevation (6.7%); grade 3 pneumonitis events (20%); grade ≥3 treatment-related adverse event 20 (60.6%); adverse events in ≥10% hypertension (15.2%)	NCT02942329
Keynote-426 (phase III, *n* = 429)	Renal cell carcinoma	Pembrolizumab	Axitinib, sunitinib	ORR 59.3%, PFS 15.1 months	Grade 3 or higher AEs 75.8%	NCT02853331
Keynote-021 (first-line, *n* = 123)	Advanced NSCLC	Pembrolizumab	Pemetrexed and carboplatin	ORR 56.7 vs. 30.2%	27% grade 3–5 treatment-related adverse events	NCT02039674

*MPFS, median progression-free survival; AEs, adverse events; TBD, to be determined; AEs, adverse events; ORR, objective response rate; GC/EGJC, gastric or esophagogastric junction cancer*.

### Combination With CTLA-4 mAbs

Cytotoxic T-lymphocyte–associated protein 4 was demonstrated to have a potent inhibitory role in Treg cell responses. Disruption of CTLA-4–CD80/CD86 interaction with anti–CTLA-4 antibody results in tumor rejection through enhancement of T-cell effector responses (58). The phase 3 trial CheckMate067 involving patients with advanced melanoma showed a significant improvement in ORR and OS in the nivolumab (PD-1 antibody) plus ipilimumab (CTLA-4 antibody) group; compared to CTLA-4 and PD-1 monotherapy, the risks of death in the combination therapy with the two drugs decreased by 46 and 35%, respectively. In the combination group, 21% of patients showed complete disappearance of the lesion and achieved complete remission, whereas 37% of the patients had 30% reduction in lesions and achieved partial remission. However, 59% of patients in the combination group reported three to four adverse events (59). In the KEYNOTE-006 study, the 5-years survival rate was 38.7 and 31% in the Keytruda combination group and in ipilimumab monotherapy, respectively (60). The combination of two immunotherapies had a much better effect, but the cost of the treatment also increased, and so did the side effects.

### Combination With Chemotherapy and Radiation

Preclinical research has suggested immunomodulatory properties for chemotherapy and irradiation (61). In 2018, the US FDA formally approved the PD-1 antibody Keytruda in combination with chemotherapy as the first line of treatment for NSCLC, regardless of the level of PD-L1 expression in patients, excluding patients with EGFR mutations and ALK fusion (62). Among the 616 patients, 410 patients received PD-1 combination therapy using Keytruda plus pemetrexed and platinum; the other 206 patients received chemotherapy with placebo plus pemetrexed and platinum. After 10.5 months of follow-up, the combination treatment group had an overwhelming advantage in all aspects. The ORR of the combined treatment group was as high as 47.6%, as compared to 18.9% in the chemotherapy group, and the combination treatment reduced the risk of disease progression by 48% (62). It is worth mentioning that regardless of the level of PD-L1 expression, the survival of patients in the combined treatment group was significantly prolonged. At the same time, many clinical trials of combination therapy with PD-1 inhibitors have yielded many exciting results.

### Combination With Targeted Therapy

Targeted drugs have high efficiency but are susceptible to develop drug resistance. Therefore, targeted drugs in combination with ICIs may achieve high efficiency and long-lasting effect. The US FDA approved the PD-1 antibody Keytruda in combination with acitretin, an oral retinoid, for the treatment of patients with advanced renal cell carcinoma (63). This is the first PD-1 antibody and targeted combination therapy, officially leading to a new era of “immunity plus targeting” cancer treatment. Phase III clinical trial Keynote-426, which recruited patients with renal cell carcinoma from multiple countries around the world to more assess the efficacy of Keytruda in combination with axitinib, which is a second-generation tyrosine kinase inhibitor that selectively inhibits vascular endothelial growth factor receptors (VEGFR-1, VEGFR-2, VEGFR-3) (64). The ORR of the pembrolizumab–axitinib group was as high as 59.3%, whereas that of the sunitinib group was only 35.7%. Sunitinib is a small-molecule multitargeted receptor tyrosine kinase inhibitor. The PFS rates were 11.1 and 15.1 months in the sunitinib group and combination group, respectively, reducing the risk of death or progression by 31%. The 12-months survival rate was 89.9 and 78.3% for the combination group and sunitinib group, respectively, and the combination treatment group reduced the risk of death by 47% (65).

### Side Effects and Resistance of PD-1/PD-L1 Blockade

An emerging challenge for PD-1/PD-L1 antibody therapy is the different levels of immune-related adverse events (irAEs), which include cardiotoxicity (66–68); cytokine release syndrome (69); myocarditis (70, 71); pneumonitis (72–74); hepatitis (74); thyroiditis (75, 76); endocrine dysfunction (77); fatigue, rash, and diarrhea (78, 79); and polymyalgia rheumatica/giant cell arteritis (80). Immune-related adverse events typically originate in the skin, gastrointestinal tract, liver, and endocrine system, although other organ systems may also be affected (81). Martins et al. (82) summarized the different types of irAEs and discussed the epidemiology and kinetics, risk factors, subtypes, pathophysiology, and screening and monitoring strategies for these adverse events and highlighted the important effects of managing irAEs. There are currently several guides on the management of irAEs (83–85).

Although immunotherapy is a long-acting method, it also develops resistance. After 21 months of follow-up in a study involving 205 patients with effective use of Keytruda, 74% of patients were still effective, whereas 26% developed resistance (86). Similarly, after a long-term follow-up of 42 patients with PD-1–effective malignant melanoma, 15 had developed resistance, and the resistance rate was 35% (87). CMP-001 (Checkmate Pharmaceuticals, Lianyungang, China), an investigational TLR9 agonist, could potentially mitigate this resistance, with an effective rate as high as 22%, including partial tumor disappearance (88).

In addition to FDA-approved methods, there are many potential discoveries that have yielded promising results in preclinical studies. Tumor necrosis factor (TNF) is upregulated in the intestine of patients suffering from colitis after dual ipilimumab and nivolumab immunotherapy. In the artificially induced mouse inflammatory bowel disease model, the anti–CTLA-4 and anti–PD-1 double treatment would aggravate inflammation. Prophylactic anti-TNF antibody or TNF inhibitor (TNFR2–immunoglobulin G) can significantly reduce the increased colitis caused by double-checkpoint blockade. Meanwhile, the authors found that TNF blockade could further enhance the infiltration of tumor-specific CD8^+^ T cells into the tumor microenvironment and draining lymph nodes caused by anti–PD-1 and anti–CTLA-4 monoclonal antibody combination therapy (89). A phase I investigator-initiated trial testing the safety of this combined approach is currently in progress.

## Concluding Remarks

Programmed death 1/PD-L1 antibodies have become the standard treatment for more than 10 kinds of tumors. Currently, nine such antibodies have been approved for marketing worldwide, and still a large number of clinical trials are assessing the other therapeutic potential of PD-1/PD-L1 antibodies as a single drug or combination. The existence of individual heterogeneity has brought great difficulties to predict the prognosis or survival of patients treated with ICIs through a single diagnostic indicator. New markers or groups that integrate multiple information are needed to predict the efficacy of anti–PD-1/PD-L1 immunotherapy. Immunotherapy requires real-time tracking of immune cells and evaluation of tumor responsiveness to immunotherapy by non-intrusive detection methods. Currently, researchers use biopsy or surgical resection of tumor samples for detection, but if relevant test factors can be found in the blood, it would be of great significance in determining the clinical effectiveness and responsiveness of immunotherapy. *In vitro* research models need to be closer to the state of the body, such as organoids incorporating the immune microenvironment (89, 90). Meanwhile, organoids can better reveal tumor progression and drug resistance and contribute to anticancer drug screening in the future (90).

Immunotherapy opens the way for precise treatment of malignant tumors. The current problems that ICIs need to overcome include accurate screening of sensitive populations, development of real-time dynamic monitoring of curative effects, accompanying diagnostic techniques and methods, development of high-throughput multimolecular multifactor integrated analysis methods, and sequential treatment. The key to improving PD-1 blockade therapy is the development of combination therapy, including other checkpoints of the T cells or other immune cells, such as CTLA-4 and NKG2D (91). Further, molecular markers, including PD-L1, TMB, MSI, and TIL, need to be explored to guide clinical treatment. Next steps in immuno-oncology: enhancing antitumor effects through appropriate patient selection and rationally designed combination strategies. The application of single-cell sequencing technology reveals the heterogeneity of the tumor microenvironment and can help in the better understanding of tumor development and immune evasion.

## Author Contributions

YJ drafted the manuscript. XZ reviewed the manuscript structure and ideas. JF evaluated and reviewed manuscript structure, ideas, and science. HW conceived the topic and revised the manuscript. All authors read and approved the final manuscript.

### Conflict of Interest

The authors declare that the research was conducted in the absence of any commercial or financial relationships that could be construed as a potential conflict of interest.

## References

[1] Pardoll DM. The blockade of immune checkpoints in cancer immunotherapy. Nat Rev Cancer. (2012) 12:252–64. 10.1038/nrc3239 22437870 PMC4856023

[2] Freeman GJ Long AJ Iwai Y Bourque K Chernova T Nishimura H. Engagement of the PD-1 immunoinhibitory receptor by a novel B7 family member leads to negative regulation of lymphocyte activation. J Exp Med. (2000) 192:1027–34. 10.1084/jem.192.7.1027 11015443 PMC2193311

[3] Korman AJ Peggs KS Allison JP. Checkpoint blockade in cancer immunotherapy. Adv Immunol. (2006) 90:297–339 10.1016/s0065-2776(06)90008-x 16730267 PMC1951510

[4] Gajewski TF Schreiber H Fu YX. Innate and adaptive immune cells in the tumor microenvironment. Nat Immunol. (2013) 14:1014–22. 10.1038/ni.2703 24048123 PMC4118725

[5] Miao D Margolis CA Gao W Voss MH Li W Martini DJ. Genomic correlates of response to immune checkpoint therapies in clear cell renal cell carcinoma. Science. (2018) 359:801–06. 10.1126/science.aan5951 29301960 PMC6035749

[6] Le DT Durham JN Smith KN Wang H Bartlett BR Aulakh LK. Mismatch repair deficiency predicts response of solid tumors to PD-1 blockade. Science. (2017) 357:409–13. 10.1126/science.aan6733 28596308 PMC5576142

[7] Samstein RM Lee CH Shoushtari AN Hellmann MD Shen R Janjigian YY. Tumor mutational load predicts survival after immunotherapy across multiple cancer types. Nat Genet. (2019) 51:202–6. 10.1038/s41588-018-0312-8 30643254 PMC6365097

[8] Mandal R Samstein RM Lee KW Havel JJ Wang H Krishna C. Genetic diversity of tumors with mismatch repair deficiency influences anti-PD-1 immunotherapy response. Science. (2019) 364:485–91. 10.1126/science.aau0447 31048490 PMC6685207

[9] Van Allen EM Miao D Schilling B Shukla SA Blank C Zimmer L. Genomic correlates of response to CTLA-4 blockade in metastatic melanoma. Science. (2015) 350:207–11. 10.1126/science.aad0095 26359337 PMC5054517

[10] Rizvi NA Hellmann MD Snyder A Kvistborg P Makarov V Havel JJ. Cancer immunology. Mutational landscape determines sensitivity to PD-1 blockade in non-small cell lung cancer. Science. (2015) 348:124–8. 10.1126/science.aaa1348 25765070 PMC4993154

[11] Snyder A Makarov V Merghoub T Yuan J Zaretsky JM Desrichard A. Genetic basis for clinical response to CTLA-4 blockade in melanoma. N Engl J Med. (2014) 371:2189–99. 10.1056/NEJMoa1406498 25409260 PMC4315319

[12] Yarchoan M Hopkins A Jaffee EM. Tumor mutational burden and response rate to PD-1 inhibition. N Engl J Med. (2017) 377:2500–1. 10.1056/NEJMc1713444 29262275 PMC6549688

[13] Le DT Uram JN Wang H Bartlett BR Kemberling H Eyring AD. PD-1 blockade in tumors with mismatch-repair deficiency. N Engl J Med. (2015) 372:2509–20. 10.1056/NEJMc1510353 26028255 PMC4481136

[14] Zou W Chen L. Inhibitory B7-family molecules in the tumour microenvironment. Nat Rev Immunol. (2008) 8:467–77. 10.1038/nri2326 18500231

[15] Thompson RH Kuntz SM Leibovich BC Dong H Lohse CM Webster WS. Tumor B7-H1 is associated with poor prognosis in renal cell carcinoma patients with long-term follow-up. Cancer Res. (2006) 66:3381–5. 10.1158/0008-5472.CAN-05-4303 16585157

[16] Rosenberg JE Hoffman-Censits J Powles T van der Heijden MS Balar AV Necchi A. Atezolizumab in patients with locally advanced and metastatic urothelial carcinoma who have progressed following treatment with platinum-based chemotherapy: a single-arm, multicentre, phase 2 trial. Lancet. (2016) 387:1909–20. 10.1016/s0140-6736(16)00561-4 26952546 PMC5480242

[17] Rossi A Noia VD Gkountakos A D'Argento E Sartori G Vita E. PD-L1 for selecting non-small-cell lung cancer patients for first-line immuno-chemotherapy combination: a systematic review and meta-analysis. Immunotherapy. (2019) 11:921–30 10.2217/imt-2018-0198 31155995

[18] Garon EB Rizvi NA Hui R Leighl N Balmanoukian AS Eder JP. Pembrolizumab for the treatment of non–small-cell lung cancer. N Engl J Med. (2015) 372:2018–28. 10.1056/NEJMoa1501824 25891174

[19] Borghaei H Paz-Ares L Horn L Spigel DR Steins M Ready NE Nivolumab versus docetaxel in advanced non-squamous non-small-cell lung cancer. N Engl J Med. (2015) 373:1627–39. 10.1056/NEJMoa1507643 26412456 PMC5705936

[20] Ferris RL Blumenschein G Fayette J Guigay J Colevas AD Licitra L. Nivolumab for recurrent squamous-cell carcinoma of the head and neck. N Engl J Med. (2016) 375:1856–67. 10.1056/NEJMoa1602252 27718784 PMC5564292

[21] Gettinger S Horn L Jackman D Spigel D Antonia S Hellmann M. Five-year follow-up of nivolumab in previously treated advanced non-small-cell lung cancer: results from the CA209-003 study. J Clin Oncol. (2018) 36:1675–84. 10.1200/jco.2017.77.0412 29570421

[22] Brahmer J Reckamp KL Baas P Crinò L Eberhardt WE Poddubskaya E. Nivolumab versus docetaxel in advanced squamous-cell non–small-cell lung cancer. N Engl J Med. (2015) 373:123–35. 10.1056/NEJMoa1504627 26028407 PMC4681400

[23] Mahoney KM Atkins M.B. Prognostic and predictive markers for the new immunotherapies. Oncology. (2014) 28(Suppl.3):39–48. 25384886

[24] Topalian SL Taube JM Anders RA Pardoll DM. Mechanism-driven biomarkers to guide immune checkpoint blockade in cancer therapy. Nat Rev Cancer. (2016) 16:275–87. 10.1038/nrc.2016.36 27079802 PMC5381938

[25] Havel JJ Chowell D Chan TA. The evolving landscape of biomarkers for checkpoint inhibitor immunotherapy. Nat Rev Cancer. (2019) 19:133–50. 10.1038/s41568-019-0116-x 30755690 PMC6705396

[26] Gibney GT Weiner LM Atkins MB. Predictive biomarkers for checkpoint inhibitor-based immunotherapy. Lancet Oncol. (2016) 17:e542–51. 10.1016/S1470-2045(16)30406-5 27924752 PMC5702534

[27] Chen G Huang AC Zhang W Zhang G Wu M Xu W. Exosomal PD-L1 contributes to immunosuppression and is associated with anti-PD-1 response. Nature. (2018) 560:382–6. 10.1038/s41586-018-0392-8 30089911 PMC6095740

[28] Poggio M Hu T Pai CC Chu B Belair CD Chang A. Suppression of exosomal PD-L1 induces systemic anti-tumor immunity and memory. Cell. (2019) 177:414–27.e13. 10.1016/j.cell.2019.02.016 30951669 PMC6499401

[29] Del Re M Marconcini R Pasquini G Rofi E Vivaldi C Bloise F. PD-L1 mRNA expression in plasma-derived exosomes is associated with response to anti-PD-1 antibodies in melanoma and NSCLC. Br Cancer J. (2018) 118:820–4. 10.1038/bjc.2018.9 29509748 PMC5886129

[30] Yearley JH Gibson C Yu N Moon C Murphy E Juco J. PD-L2 expression in human tumors: relevance to anti-PD-1 therapy in cancer. Clin Cancer Res. (2017) 23:3158–67. 10.1158/1078-0432.CCR-16-1761 28619999

[31] Zilionis R Engblom C Pfirschke C Savova V Zemmour D Saatcioglu HD. Single-cell transcriptomics of human and mouse lung cancers reveals conserved myeloid populations across individuals and species. Immunity. (2019) 50:1317–34.e10. 10.1016/j.immuni.2019.03.009 30979687 PMC6620049

[32] Fridman WH Zitvogel L Sautes-Fridman C Kroemer G. The immune contexture in cancer prognosis and treatment. Nat Rev Clin Oncol. (2017) 14:717–34. 10.1038/nrclinonc.2017.101 28741618

[33] Aizarani N Saviano A Sagar Mailly L Durand S Herman JS. A human liver cell atlas reveals heterogeneity and epithelial progenitors. Nature. (2019) 572:199–204. 10.1038/s41586-019-1373-2 31292543 PMC6687507

[34] Fridman WH Pagès F Sautès-Fridman C Galon J. The immune contexture in human tumours: impact on clinical outcome. Nat Rev Cancer. (2012) 12:298–306. 10.1038/nrc3245 22419253

[35] Barry KC Hsu J Broz ML Cueto FJ Binnewies M Combes AJ. A natural killer–dendritic cell axis defines checkpoint therapy–responsive tumor microenvironments. Nat Med. (2018) 24:1178–91. 10.1038/s41591-018-0085-8 29942093 PMC6475503

[36] Neubert NJ Schmittnaegel M Bordry N Nassiri S Wald N Martignier C Speiser: T cell-induced CSF1 promotes melanoma resistance to PD1 blockade. Sci Transl Med. (2018) 10: eaan3311 10.1126/scitranslmed.aan3311 29643229 PMC5957531

[37] Arlauckas SP Garris CS Kohler RH Kitaoka M Cuccarese MF Yang KS. *In vivo* imaging reveals a tumor-associated macrophage-mediated resistance pathway in anti-PD-1 therapy. Sci Transl Med. (2017) 9:eaal3604. 10.1126/scitranslmed.aal3604 28490665 PMC5734617

[38] Gallagher EJ LeRoith D. Obesity and diabetes: the increased risk of cancer and cancer-related mortality. Physiol Rev. (2015) 95:727–48. 10.1152/physrev.00030.2014 26084689 PMC4491542

[39] Gregor MF Hotamisligil GS. Inflammatory mechanisms in obesity. Annu Rev Immunol. (2011) 29:415–45. 10.1146/annurev-immunol-031210-101322 21219177

[40] Wang Z Aguilar EG Luna JI Dunai C Khuat LT Le CT. Paradoxical effects of obesity on T cell function during tumor progression and PD-1 checkpoint blockade. Nat Med. (2019) 25:141–51. 10.1038/s41591-018-0221-5 30420753 PMC6324991

[41] McQuade JL Daniel CR Hess KR Mak C Wang DY Rai RR. Association of body-mass index and outcomes in patients with metastatic melanoma treated with targeted therapy, immunotherapy, or chemotherapy: a retrospective, multicohort analysis. Lancet Oncol. (2018) 19:310–22. 10.1016/S1470-2045(18)30078-0 29449192 PMC5840029

[42] Murphy WJ Longo DL. The surprisingly positive association between obesity and cancer immunotherapy efficacy. JAMA. (2019) 321:1247–1248. 10.1001/jama.2019.0463 30882850

[43] McQuade JL Daniel CR Hess KR Mak C Wang DY Rai RR. Association of body-mass index and outcomes in patients with metastatic melanoma treated with targeted therapy, immunotherapy, or chemotherapy: a retrospective, multicohort analysis. Lancet Oncol. (2018) 19:310–22. 10.1016/s1470-2045(18)30078-0 29449192 PMC5840029

[44] Cortellini A Bersanelli M Buti S Cannita K Santini D Perrone F. A multicenter study of body mass index in cancer patients treated with anti-PD-1/PD-L1 immune checkpoint inhibitors: when overweight becomes favorable. J Immunother Cancer. (2019) 7:57. 10.1186/s40425-019-0527-y 30813970 PMC6391761

[45] Kichenadasse G Miners JO Mangoni AA Rowland A Hopkins AM Sorich MJ. Association between body mass index and overall survival with immune checkpoint inhibitor therapy for advanced non-small cell lung cancer. JAMA Oncol. (2019). 10.1001/jamaoncol.2019.5241. [Epub ahead of print]. 31876896 PMC6990855

[46] Sivan A Corrales L Hubert N Williams JB Aquino-Michaels K Earley ZM. Commensal *Bifidobacterium* promotes antitumor immunity and facilitates anti-PD-L1 efficacy. Science. (2015) 350:1084–9. 10.1126/science.aac4255 26541606 PMC4873287

[47] Vétizou M Pitt JM Daillère R Lepage P Waldschmitt N Flament C. Anticancer immunotherapy by CTLA-4 blockade relies on the gut microbiota. Science. (2015) 350:1079–84. 10.1126/science.aad1329 26541610 PMC4721659

[48] Gopalakrishnan V Spencer CN Nezi L Reuben A Andrews MC Karpinets TV. Gut microbiome modulates response to anti-PD-1 immunotherapy in melanoma patients. Science. (2018) 359:97–103. 10.1126/science.aan4236 29097493 PMC5827966

[49] Diem S Schmid S Krapf M Flatz L Born D Jochum W. Neutrophil-to-Lymphocyte ratio (NLR) and Platelet-to-Lymphocyte ratio (PLR) as prognostic markers in patients with non-small cell lung cancer (NSCLC) treated with nivolumab. Lung Cancer. (2017) 111:176–81. 10.1016/j.lungcan.2017.07.024 28838390

[50] Demirci NS Erdem GU. Prognostic role of neutrophil-to-lymphocyte ratio (NLR) in patients with operable ampullary carcinoma. Bosn J Basic Med Sci. (2018) 18:268–74. 10.17305/bjbms.2017.2530 29131967 PMC6087558

[51] Liao LJ Hsu WL Wang CT Lo WC Cheng PW Shueng PW Prognostic impact of pre-treatment neutrophil-to-lymphocyte ratio (NLR) in nasopharyngeal carcinoma: a retrospective study of 180 Taiwanese patients. Clin Otolaryngol. (2018) 43:463–9. 10.1111/coa.12992 28950051

[52] Ethier JL Desautels D Templeton A Shah PS Amir E. Prognostic role of neutrophil-to-lymphocyte ratio in breast cancer: a systematic review and meta-analysis. Breast Cancer Res. (2017) 19:2. 10.1186/s13058-016-0794-1 28057046 PMC5217326

[53] Chen N Liu S Huang L Li W Yang W Cong T. Prognostic significance of neutrophil-to-lymphocyte ratio in patients with malignant pleural mesothelioma: a meta-analysis. Oncotarget. (2017) 8:57460–9. 10.18632/oncotarget.15404 28915685 PMC5593657

[54] Ameratunga M Chénard-Poirier M Moreno Candilejo I Pedregal M Lui A Dolling D. Neutrophil-lymphocyte ratio kinetics in patients with advanced solid tumours on phase I trials of PD-1/PD-L1 inhibitors. Eur Cancer J. (2018) 89:56–63. 10.1016/j.ejca.2017.11.012 29227818

[55] Suh KJ Kim SH Kim YJ Kim M Keam B Kim TM. Post-treatment neutrophil-to-lymphocyte ratio at week 6 is prognostic in patients with advanced non-small cell lung cancers treated with anti-PD-1 antibody. Cancer Immunol Immunother. (2018) 67:459–70. 10.1007/s00262-017-2092-x 29204702 PMC11028357

[56] Xu J Zhang Y Jia R Yue C Chang L Liu R. Anti-PD-1 antibody SHR-1210 combined with apatinib for advanced hepatocellular carcinoma, gastric, or esophagogastric junction cancer: an open-label, dose escalation and expansion study. Clin Cancer Res. (2019) 25:515–23. 10.1158/1078-0432.Ccr-18-2484 30348638

[57] Yu JX Hodge JP Oliva C Neftelinov ST Hubbard-Lucey VM Tang J Trends in clinical development for PD-1/PD-L1 inhibitors. Nat Rev Drug Discov. (2019). 10.1038/d41573-019-00182-w. [Epub ahead of print]. 32127660

[58] Buchbinder EI Desai A. CTLA-4 and PD-1 pathways: similarities, differences, and implications of their inhibition. Am J Clin Oncol. (2016) 39:98–106. 10.1097/coc.0000000000000239 26558876 PMC4892769

[59] Hodi FS Chiarion-Sileni V Gonzalez R Grob JJ Rutkowski P Cowey CL. Nivolumab plus ipilimumab or nivolumab alone versus ipilimumab alone in advanced melanoma (CheckMate 067): 4-year outcomes of a multicentre, randomised, phase 3 trial. Lancet Oncol. (2018) 19:1480–92. 10.1016/s1470-2045(18)30700-9 30361170

[60] Robert C Ribas A Schachter J Arance A J.-Grob J Mortier L. Pembrolizumab versus ipilimumab in advanced melanoma (KEYNOTE-006): post-hoc 5-year results from an open-label, multicentre, randomised, controlled, phase 3 study. Lancet Oncol. (2019) 20:P1239–51. 10.1016/s1470-2045(19)30388-2 31345627

[61] Voorwerk L Slagter M Horlings HM Sikorska K van de Vijver KK de Maaker M Immune induction strategies in metastatic triple-negative breast cancer to enhance the sensitivity to PD-1 blockade: the TONIC trial. Nat Med. (2019) 25:920–8 10.1038/s41591-019-0432-4 31086347

[62] Frederickson AM Arndorfer S Zhang I Lorenzi M Insinga R Arunachalam A. Pembrolizumab plus chemotherapy for first-line treatment of metastatic nonsquamous non-small-cell lung cancer: a network meta-analysis. Immunotherapy. (2019) 11:407–28. 10.2217/imt-2018-0193 30712477

[63] Pembrolizumab (KEYTRUDA®) in combination with carboplatin-paclitaxel/Nab-paclitaxel for metastatic squamous non small cell lung cancer, first line (2019). Available online at: https://www.fda.gov/drugs/fda-approves-pembrolizumab-combination-chemotherapy-first-line-treatment-metastatic-squamous-nsclc

[64] Atkins MB Plimack ER Puzanov I Fishman MN McDermott DF Cho DC. Axitinib in combination with pembrolizumab in patients with advanced renal cell cancer: a non-randomised, open-label, dose-finding, and dose-expansion phase 1b trial. Lancet Oncol. (2018) 19:405–15. 10.1016/s1470-2045(18)30081-0 29439857 PMC6860026

[65] Rini BI Plimack ER Stus V Gafanov R Hawkins R Nosov D. Pembrolizumab plus axitinib versus sunitinib for advanced renal-cell carcinoma. N Engl J Med. 380:1116–27 (2019) 10.1056/NEJMoa1816714 30779529

[66] Varricchi G Galdiero MR Marone G Criscuolo G Triassi M Bonaduce D. Cardiotoxicity of immune checkpoint inhibitors. ESMO Open. (2017) 2:e000247. 10.1136/esmoopen-2017-000247 29104763 PMC5663252

[67] Varricchi G Marone G Mercurio V Galdiero MR Bonaduce D Tocchetti CG. Immune checkpoint inhibitors and cardiac toxicity: an emerging issue. Curr Med Chem. (2018) 25:1327–39. 10.2174/0929867324666170407125017 28403786

[68] Zhang JC Chen WD Alvarez JB Jia K Shi L Wang Q. Cancer immune checkpoint blockade therapy and its associated autoimmune cardiotoxicity. Acta Pharmacol Sin. (2018) 39:1693–8. 10.1038/s41401-018-0062-2 29991709 PMC6289335

[69] Rotz SJ Leino D Szabo S Mangino JL Turpin BK Pressey JG. Severe cytokine release syndrome in a patient receiving PD-1-directed therapy. Pediatr Blood Cancer. (2017) 64:e26642. 10.1002/pbc.26642 28544595

[70] Moslehi JJ Salem JE Sosman JA Lebrun-Vignes B Johnson DB. Increased reporting of fatal immune checkpoint inhibitor-associated myocarditis. Lancet. (2018) 391:933. 10.1016/S0140-6736(18)30533-6 29536852 PMC6668330

[71] Tadokoro T Keshino E Makiyama A Sasaguri T Ohshima K Katano H. Acute lymphocytic myocarditis with anti-pd-1 antibody nivolumab. Circ Heart Fail. (2016) 9:e003514. 10.1161/circheartfailure.116.003514 27650418

[72] Nishino M Chambers ES Chong CR Ramaiya NH Gray SW Marcoux JP. Anti-PD-1 Inhibitor-Related Pneumonitis in Non-Small Cell Lung Cancer. Cancer Immunol Res. (2016) 4:289–93. 10.1158/2326-6066.Cir-15-0267 26865455 PMC4818710

[73] Khunger M Rakshit S Pasupuleti V Hernandez AV Mazzone P Stevenson J. Incidence of pneumonitis with use of programmed death 1 and programmed death-ligand 1 inhibitors in non-small cell lung cancer: a systematic review and meta-analysis of trials. Chest. (2017) 152:271–281. 10.1016/j.chest.2017.04.177 28499515

[74] Wang DY Salem JE Cohen JV Chandra S Menzer C Ye F. Fatal toxic effects associated with immune checkpoint inhibitors: a systematic review and meta-analysis. JAMA Oncol. (2018) 4:1721–8. 10.1001/jamaoncol.2018.3923 30242316 PMC6440712

[75] Delivanis DA Gustafson MP Bornschlegl S Merten MM Kottschade L Withers S. Pembrolizumab-induced thyroiditis: comprehensive clinical review and insights into underlying involved mechanisms. J Clin Endocrinol Metab. (2017) 102:2770–80. 10.1210/jc.2017-00448 28609832 PMC5546861

[76] Yamauchi I Sakane Y Fukuda Y Fujii T Taura D Hirata M. Clinical features of nivolumab-induced thyroiditis: a case series study. Thyroid. (2017) 27:894–901. 10.1089/thy.2016.0562 28537531

[77] Barroso-Sousa R Barry WT Garrido-Castro AC Hodi FS Min L Krop IE. Incidence of endocrine dysfunction following the use of different immune checkpoint inhibitor regimens: a systematic review and meta-analysis. JAMA Oncol. (2018) 4:173–82. 10.1001/jamaoncol.2017.3064 28973656 PMC5838579

[78] Larkin J Chiarion-Sileni V Gonzalez R Grob JJ Cowey CL Lao CD Combined nivolumab and ipilimumab or monotherapy in untreated melanoma. N Engl J Med. (2015) 373:23–34. 10.1056/NEJMoa1504030 26027431 PMC5698905

[79] Weber JS D'Angelo SP Minor D Hodi FS Gutzmer R Neyns B. Nivolumab versus chemotherapy in patients with advanced melanoma who progressed after anti-CTLA-4 treatment (CheckMate 037): a randomised, controlled, open-label, phase 3 trial. Lancet Oncol. (2015) 16:375–84. 10.1016/s1470-2045(15)70076-8 25795410

[80] Goldstein BL Gedmintas L Todd DJ. Drug-associated polymyalgia rheumatica/giant cell arteritis occurring in two patients after treatment with ipilimumab, an antagonist of ctla-4. Arthritis Rheumatol. (2014) 66:768–9. 10.1002/art.38282 24574239

[81] Weber JS Postow M Lao CD Schadendorf D. Management of adverse events following treatment with anti-programmed death-1 agents. Oncologist. (2016) 21:1230–40. 10.1634/theoncologist.2016-0055 27401894 PMC5061539

[82] Martins F Sofiya L Sykiotis GP Lamine F Maillard M Fraga M. Adverse effects of immune-checkpoint inhibitors: epidemiology, management and surveillance. Nat Rev Clin Oncol. (2019) 16:563–80. 10.1038/s41571-019-0218-0 31092901

[83] Haanen J Carbonnel F Robert C Kerr KM Peters S Larkin J. Management of toxicities from immunotherapy: ESMO Clinical Practice Guidelines for diagnosis, treatment and follow-up. Ann Oncol. (2017) 28(Suppl.4):iv119–42. 10.1093/annonc/mdx225 28881921

[84] Puzanov I Diab A Abdallah K Bingham CO Brogdon C Dadu R. Managing toxicities associated with immune checkpoint inhibitors: consensus recommendations from the Society for Immunotherapy of Cancer (SITC) Toxicity Management Working Group. J Immunother Cancer. (2017) 5:95. 10.1186/s40425-017-0300-z 29162153 PMC5697162

[85] Martins F Sykiotis GP Maillard M Fraga M Ribi C Kuntzer T. New therapeutic perspectives to manage refractory immune checkpoint-related toxicities. Lancet Oncol. (2019) 20:e54–64. 10.1016/S1470-2045(18)30828-3 30614479

[86] Ribas A Hamid O Daud A Hodi FS Wolchok JD Kefford R. Association of Pembrolizumab With Tumor Response and Survival Among Patients With Advanced Melanoma. JAMA. (2016) 315:1600–9. 10.1001/jama.2016.4059 27092830

[87] Zaretsky JM Garcia-Diaz A Shin DS Escuin-Ordinas H Hugo W Hu-Lieskovan S. Mutations associated with acquired resistance to pd-1 blockade in melanoma. N Engl J Med. (2016) 375:819–29. 10.1056/NEJMoa1604958 27433843 PMC5007206

[88] Jesse M Zaretsky BS Garcia-Diaz A Daniel S Shin MD Helena Escuin-Ordinas Warming “Cold” Melanoma with TLR9 Agonists. Cancer Discov. (2018) 8:670 10.1158/2159-8290.Cd-nd2018-004 29669722

[89] Lee SH Hu W Matulay JT Silva MV Owczarek TB Kim K. Tumor Evolution and Drug Response in Patient-Derived Organoid Models of Bladder Cancer. Cell. (2018) 173:515–28.e17. 10.1016/j.cell.2018.03.017 29625057 PMC5890941

[90] Fong ELS Toh TB Lin QXX Liu Z Hooi L Mohd Abdul Rashid MB. Generation of matched patient-derived xenograft *in vitro-in vivo* models using 3D macroporous hydrogels for the study of liver cancer. Biomaterials. (2018) 159:229–40. 10.1016/j.biomaterials.2017.12.026 29353739

[91] Zhang Q Bi J Zheng X Chen Y Wang H Wu W. Blockade of the checkpoint receptor TIGIT prevents NK cell exhaustion and elicits potent anti-tumor immunity. Nat Immunol. (2018) 19:723–32. 10.1038/s41590-018-0132-0 29915296

